# Apatinib Combined With SOX Regimen in Conversion Treatment of Advanced Gastric Cancer: A Case Series and Literature Review

**DOI:** 10.3389/fphar.2020.01027

**Published:** 2020-07-14

**Authors:** Dingyi Jiang, Yunyun Xu, Yunwang Chen, Jiahong Jiang, Mingxing Wang, Min Yang, Zheling Chen, Liu Yang

**Affiliations:** ^1^Department of Medical Oncology, The Qingdao University Medical College, Qingdao, China; ^2^Department of Medical Oncology, Zhejiang Provincial People’s Hospital, People’s Hospital of Hangzhou Medical College, Hangzhou, China; ^3^Department of Medical Oncology, Bengbu Medical College, Bengbu, China

**Keywords:** apatinib, gastric cancer, conversion therapy, case, review

## Abstract

Gastric cancer is a common digestive tract tumor and the second most prevalent cancer. The prognosis of advanced gastric cancer is poor. Conversion therapy can reduce tumor burden, downgrade tumor, and increase the possibility of complete resection, thus prolonging the survival time of patients with gastric cancer. In conversion therapy, chemotherapy and targeted therapy are the main methods of medical treatment, which can control tumor growth and recurrence. As an antiangiogenic targeted drug, apatinib is widely used in the third-line treatment of advanced gastric cancer. Recent studies have shown that it may be of great help in rapid reduction of tumor stage and improvement of prognosis in conversion therapy. This study reported three cases of gastric cancer complicated with multiple abdominal and retroperitoneal lymph node metastases. After receiving apatinib combined with SOX regimen for four cycles, computed tomography showed that the focus and lymph node metastasis were reduced after treatment, and primary tumors were resected. Postoperative pathology result showed that the patients got R0 resection. After radical surgery, the maintenance therapy including apatinib was given. The progression-free survival time was more than 10 months. Apatinib combined with SOX regimen as a conversion therapy for advanced gastric adenocarcinoma increases the possibility of successful surgical resection, which might prolong the survival time of patients.

## Introduction

According to the latest Estimating the global cancer incidence and mortality in 2018, the incidence of gastric cancer ranks sixth and mortality ranks second (Ferlay et al., 2019). Many patients are already in the advanced stage of tumor when they are diagnosed for the first time, and the prognosis of patients with advanced tumor is poor (An et al., 2009). The median survival time of untreated advanced gastric cancer is less than 1 year. For metastatic gastric cancer, many studies are exploring possible posterior line treatment regimen to improve the survival rate of patients (Yoshida et al., 2006; Koizumi et al., 2008; Bang et al., 2010; Tanabe et al., 2010; Koizumi et al., 2014), However, the survival time of the patients is still short. In order to prolong the survival time of patients and improve their quality of life, the research on new treatment schemes still needs to be accelerated (Shen et al., 2013). The transformation therapy of gastric cancer is a hot topic recently, and its purpose is to achieve R0 resection after comprehensive treatment of tumors that are unresectable or slightly resectable for technical or oncological reasons (Yoshida et al., 2016).

In recent years, studies by Sato Y et al. and Yamaguchi K et al. have shown that conversion therapy can achieve a relatively high remission rate and surgical conversion rate, and improve the long-term survival of patients (Sato et al., 2017; Yamaguchi et al., 2018). Studies by Fukuchi et al. and Einama et al. also suggest that conversion therapy can improve the survival of patients with initial unresectable gastric cancer (Einama et al., 2017; Fukuchi et al., 2017). Multiple studies have shown that the treatment effect of advanced gastric cancer has not been significantly improved. The median survival time has been hovering between 10 and 14 months (Koizumi et al., 2008; Bang et al., 2010; Ito et al., 2017). In conversion therapy, the choice of drugs and treatment is equally critical. In the past clinical studies, the fluorouracil, platinum, and paclitaxel drugs were used for conversion therapy in advanced gastric cancer. On this basis, a combination of different drugs was carried out. Some retrospective clinical research results suggest that S-1 based combination chemotherapy benefits patients during the conversion treatment phase. A small sample clinical study involving 18 patients showed that the combined treatment with paclitaxel and S-1 resulted in a median survival of 64 months, with an R0 resection rate of 72% (Ishigami et al., 2008). Another study on cisplatin combined with S-1 showed that the conversion therapy gave patients a median survival of 53 months (Fukuchi et al., 2015). Apatinib [AiTan™ (China); Rivoceranib^®^ (global)] is a novel small molecular inhibitor that selectively acts on VEGFR-2 tyrosine kinase (Scott, 2018). It is the second antiangiogenic targeted drug approved by China for the treatment of advanced metastatic gastric cancer, mainly used in third-line therapy (Aoyama and Yoshikawa, 2016). Pharmacology shows that apatinib combined with commonly used chemotherapeutic drugs (such as oxaliplatin, 5-fluorouracil, paclitaxel, etc.) can inhibit the growth of several human tumors (including lung cancer, gastric cancer, colorectal cancer, etc.) in a dose-dependent manner. The anti-tumor effect of combined therapy is stronger than that of any single drug (Tian et al., 2011). The efficacy of oral apatinib in adult patients with advanced or metastatic gastric cancer or GEA who failed with two or more chemotherapy regimens was evaluated in a double-blind, multicenter, phase II trial (Li et al., 2013) and a randomized, double-blind, multicenter, phase III trial (Li et al., 2016). The evaluation showed that apatinib significantly prolonged the median of PFS and OS compared with placebo. At the 91st Annual Meeting of the Japan Gastric Cancer Society (JGCA 2019) held in Shizuoka, Japan on February 28, 2019, a study retrospectively analyzed 33 patients with advanced unresectable advanced gastric cancer who received treatment from 2017 to 2018. Clinical results. The report results suggest that apatinib combined with dual drugs (S-1 combined with oxaliplatin or paclitaxel) is safe and effective for the treatment of inoperable advanced gastric cancer, and the conversion rate of R0 surgery reaches 63.6% (Yang et al., 2019).

Based on the above research results and the status of transformational therapy in gastric cancer, we explore whether apatinib can be used in the stage of transformational therapy, so as to achieve the purpose of rapid tumor retraction. In this study, apatinib combined with S-1 and oxaliplatin (SOX) regimen was performed before operation, the tumor was resected at R0, and good clinical results were achieved. This study was approved by the ethical committee at Zhejiang Provincial People’s Hospital (No. 2017KY012). The written informed consent of the patient was obtained, and any accompanying images will be published.

## Case Reports

### Case 1

**Figure 1:**
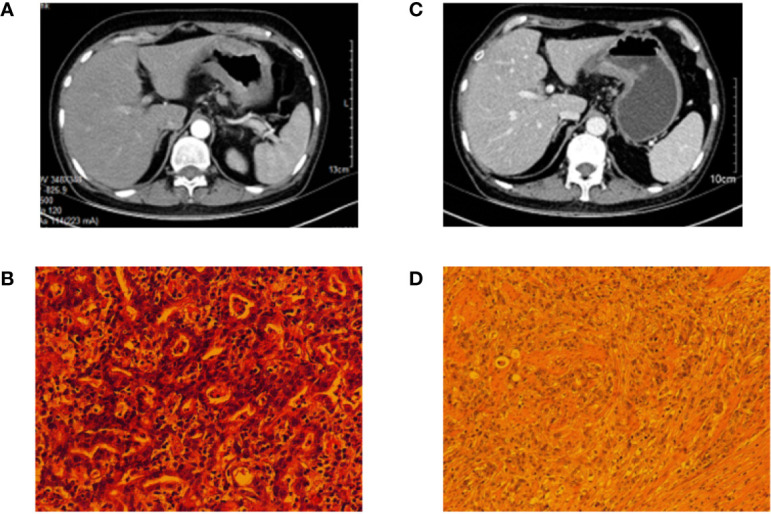
Pathology and imaging data of patient 1. **(A)** The CT of the patient at the first examination; **(B)** The gastroscopic pathology that suggested adenocarcinoma of the gastric body. **(C)** The CT of the patient after four cycles of combined chemotherapy. **(D)** Postoperative pathology of the patient.

A 56-year-old woman was admitted to the people’s Hospital of Zhejiang Province, who complained of dull pain in her upper abdomen for more than 3 months. Tumor marker results suggested that carbohydrate antigen 125 (CA12-5) was 35.2 U/ml and the carbohydrate antigen 199 (CA19-9) was 1,522 U/ml. Enhanced CT of the whole abdomen and pelvis in July 2017 suggested diffuse thickening of the gastric wall, considering the leather stomach with lymph node metastasis in the hepatogastric space (**Figure 1A**). PET-CT showed obvious thickening of gastric fundus and enlarged lymph nodes in the lesser curvature of the stomach. Gastroscopic pathology revealed adenocarcinoma of the gastric body and Helicobacter pylori (+) (**Figure 1B**). Clinical stage is T3N3M0 (Washington, 2010). MDT believes that the patient is in local advanced stage and there is no indication of operation, so it is suggested that conversion treatment should be given first. The patient received a combination of SOX regimen and apatinib. The SOX regimen included oxaliplatin 130 mg/m^2^ intravenous injection on the first day and tigio 40 mg/m2 twice a day for oral administration from the first to 14 days, repeated every 3 weeks. Apatinib 500 mg is taken orally once a day. Grade 1 neutropenia and grade 1 vomiting were observed based on the Common Terminology Criteria for Adverse Events (Chen et al., 2012). When the patient completed four cycles of combined chemotherapy, abdominal CT and PET-CT scans were performed to observe the treatment. Imaging showed that the scope of gastric malignant tumor was significantly smaller than that at the first diagnosis (**Figure 1C**), and the gastric minor curvature and hepatogastric interspace lymph nodes were significantly smaller than those at the first diagnosis. PET-CT suggested that the gastric wall of the lesser curvature of the stomach was thickened, the metabolism of FDG was increased, the scope of the lesion was significantly smaller than before, and the metabolism of FDG was decreased. The lymph nodes of the lesser curvature of the stomach were significantly reduced, and the metabolism of FDG of lymph nodes was decreased. The level of CA19-9 was 59.7U/ml and the CA125 was in normal level. Because the patient was evaluated for acceptable radical surgery, the patient was instructed to stop apatinib for 3 weeks. The patient then underwent total gastrectomy, D2 lymph node dissection, and Roux-en-Y anastomosis in November 2017. Postoperative pathology revealed diffuse poorly differentiated adenocarcinoma of the gastric body, 6 × 4 cm in size, invading the subserosa, upper and lower margin was negative with pericardial lymph nodes (1/14), lesser curved gastric lymph nodes (7/13), and suprapyloric lymph nodes (1/1) (**Figure 1D**). The pathological stage was ypT2N2M0 (Washington, 2010). Four weeks after operation, the patient received four cycles of apatinib combined with SOX at the same dose as before. After the treatment was stopped, the patients were followed up every 3 months, the last follow-up was in April 2020, and the PFS was 32 months.

### Case 2

**Figure 2:**
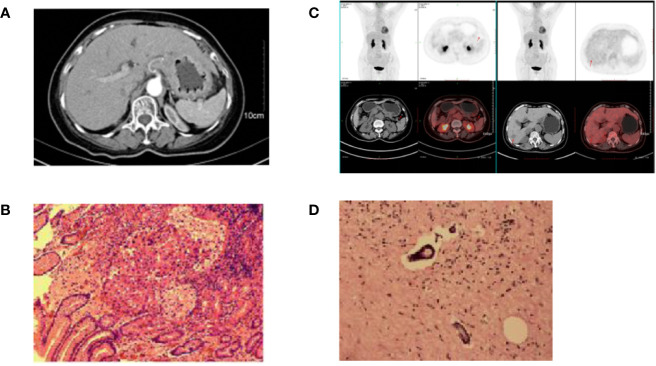
Pathology and imaging data of patient 2. **(A)** The CT of the patient at the first examination; **(B)** The gastroscopic pathology that suggested gastric poorly differentiated adenocarcinoma with abdominal lymph node metastasis; **(C)** PET-CT after four cycles of combined chemotherapy showed no focal increase in FDG in local gastric wall of gastric cardia and fundus; **(D)** Postoperative pathology of the patient.

A 68-year-old woman was admitted to the people’s Hospital of Zhejiang Province, who complained of dysphagia for half a year and aggravated for 10 days. Carcino-embryonic antigen (CEA) value was 13.2 μg/L. Enhanced CT of the whole abdomen and pelvis in April 2018 revealed thickening of the cardia and stomach floor, multiple enlarged lymph nodes around the abdominal trunk, and consideration of gastric cancer (**Figure 2A**). The pathological diagnosis was gastric poorly differentiated adenocarcinoma with abdominal lymph node metastasis (**Figure 2B**). MDT believed that the patient was in the advanced stage of gastric cancer and there was no indication of operation, so it was suggested that conversion therapy should be performed first. The patient received the combination of SOX regimen and apatinib, which was the same as before. Grade 2 neutropenia and Grade 2 vomiting were observed during treatment. When the patient completed four cycles of combined chemotherapy, a PET-CT scan was performed to observe the treatment (**Figure 2C**). PET-CT showed thicker local gastric wall of gastric cardia and fundus, no focal increase in FDG metabolism, and no increase in FDG metabolism of small perigastric lymph nodes. The complete inactivation of clinical tumor was considered and post-treatment evaluation reached CR. The CEA value was in normal level. Because the patient was evaluated for acceptable radical surgery, the patient was instructed to stop apatinib for 3 weeks. A total gastrectomy, D2 lymph node dissection, and Roux-en-Y anastomosis were performed in September 2018. Postoperative pathology showed that there was a superficial ulcerative lesion in the lesser curvature of the gastric fundus, the size was 1.7 × 1 cm, multiple intravascular tumor thrombus could be seen in the submucosa and serous layer, the subgastric resection margin was negative, and no metastasis was found in gastric lymph nodes and hepatoduodenal lymph nodes. The pathological stage was ypT2N0M0 (Washington, 2010) (**Figure 2D**). Four weeks after operation, the patient received four cycles of apatinib combined with SOX at the same dose as before. The patient developed renal insufficiency in January 2019, so she stopped treatment and was followed up closely. The last follow-up period was February 2020, and PFS was 22 months.

### Case 3

**Figure 3:**
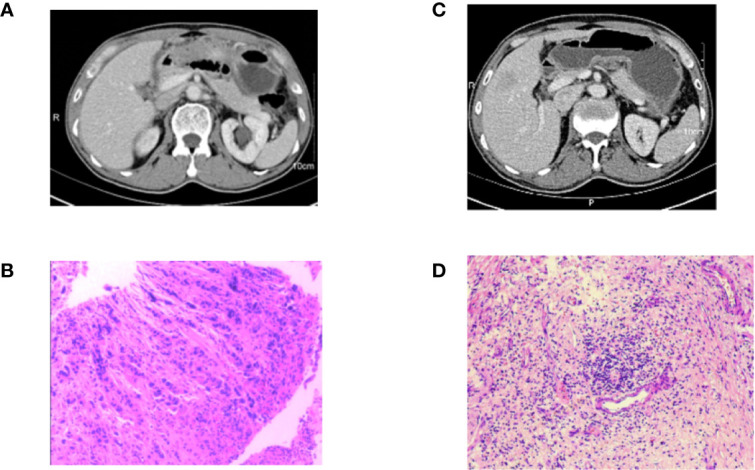
Pathology and imaging data of patient 3. **(A)** The CT of the patient at the first examination; **(B)** The gastroscopic pathology that adenocarcinoma of the lesser curvature of the gastric body; **(C)** Enhanced CT scan was performed, suggesting that the tumor was further smaller than first examination; **(D)** Postoperative pathology of the patient.

A 61-year-old man was admitted to the people’s Hospital of Zhejiang Province, who complained of abdominal pain for 2 months and aggravated for 1 week. The CA19-9 level was 91.6 U/ml. The enhancement CT of whole abdomen and pelvis in March 2017 showed that the local gastric wall of the gastric body was thickened, about 13 mm at the thickest point, with obvious enhancement, and lymph nodes seemed to be shown around it (**Figure 3A**). PET-CT showed thickening of lesser curvature and antrum, active metabolism of FDG, scattered lymphoid shadow in left sternal area, right parasternal region, septal angle, lesser curvature and retroabdominal membrane, multiple omental opacity, patchy active area of FDG metabolism, and peritoneal metastasis with malignant peritoneal effusion. Pathology revealed adenocarcinoma of the lesser curvature of the gastric body (**Figure 3B**). The clinical stage is T4N3M1 (Washington, 2010). Multi-Disciplinary Treatment (MDT) suggests that the patient with gastric cancer has systemic metastasis and has no chance of operation, so palliative chemotherapy or conversion treatment is recommended. The patient received the combination of SOX regimen and apatinib, which was the same as before. Grade 2 neutropenia and Grade 1 vomiting were observed during treatment. When the patient completed four cycles of combined chemotherapy, an enhanced CT scan was performed to observe the treatment (**Figure 3C**), suggesting that the tumor was further smaller than the third cycle, and the efficacy evaluation was PR. Thus it was suggested that the chemotherapy was continued. PET-CT examination at the end of the fifth cycle suggested no significant thickening of gastric wall and increased FDG metabolism, and no lymph node metastasis. Clinical tumor complete inactivation was considered and post-treatment evaluation reached CR. The CA19-9 level decreased to the normal level. Because the patient was evaluated for acceptable radical surgery, the patient was instructed to stop apatinib for 3 weeks. In August 2017, total gastrectomy, D2 lymph node dissection, and Roux-en-Y anastomosis were performed. The postoperative pathology showed that there was an ulcer on the lesser curved side of the gastric body, the size was about 0.8 × 0.3cm, which microscopically showed the hyperplasia of local mucous membrane, submucosa, and lamina propria myenteric fibrous tissue, the infiltration multiple lymphocyte, and no residual cancer tissue. The upper and lower cut edges were negative. Cancer cells were found in the lymph nodes of the greater curvature of the stomach (1/2), but no metastasis was found in the rest of the lymph nodes. The pathological stage is ypT1N1M0 (Washington, 2010) (**Figure 3D**). Four weeks after operation, the patient received apatinib combined with tigio maintenance treatment at the same dose as before. The patient’s ascites worsened in August 2018 and adenocarcinoma cells were found by ascites biopsy. The time of death was September 2018, 12 months for PFS and 17 months for OS.

## Discussion

Gastric cancer has become the top four malignant tumors in the world, with a high incidence in China, South Korea, Japan, and other countries (Rahman et al., 2014). Compared with Japan, China’s awareness of prevention and screening for gastric cancer is relatively weak, resulting in the majority of patients with gastric cancer often at a more advanced stage when they are diagnosed for the first time (Chen et al., 2015).

Palliative surgery, chemotherapy, radiotherapy, gastric stent, and bypass are still the main methods for the treatment of advanced gastric cancer (Izuishi and Mori, 2016). According to Japanese gastric cancer treatment guidelines 2018, For gastric cancer patients with liver or peritoneal metastasis without bleeding obstruction and other complications, tumor reduction surgery is strongly not recommended (Japanese Gastric Cancer, 2020). In contrast, ESMO Clinical Practice Guidelines suggests that gastrectomy should be considered for patients with a good response to systemic chemotherapy (Smyth et al., 2016). Previous other studies have shown that resection of primary tumors after chemotherapy is beneficial for advanced patients (Kim et al., 2011; Sougioultzis et al., 2011). Kim SW reported that after conversion therapy for the patients with gastric cancer and peritoneal implantation, the 3-year survival rate of those who can be completely resectable can reach 50% (Kim, 2014). In the clinical study of Fukuchi M et al., 40 patients with gastric cancer received conversion therapy. The 5-year survival rate of patients who received conversion therapy was 43%, and the 5-year survival rate of patients without conversion therapy was less than 1% (Fukuchi et al., 2015). In the near future, conversion therapy may become one of the main methods for the treatment of advanced gastric cancer.

Although good results have been achieved in conversion therapy, the standard of conversion therapy has not been determined. Nakajima et al. reported that the condition of patients with liver and peritoneal metastases was difficult to control after conversion treatment (Nakajima et al., 1997). Kanda et al. found that the prognosis of patients with lymph node metastasis was improved after conversion therapy, while the prognosis of patients with peritoneal metastasis was poor after conversion therapy (Kanda et al., 2012). After classifying advanced gastric cancer, Yoshida et al. believe that patients with potential resectable metastasis but no peritoneal diffusion are the best candidates for conversion therapy (Yoshida et al., 2016). In addition, the cycle of conversion therapy and the means of post-treatment evaluation are still being explored. This study showed that after four or five cycles of conversion therapy, the patients had a better chance of operation, and all the patients were evaluated by PET-CT after conversion therapy, and the metabolic level of the lesions changed, which better supported the efficacy of chemotherapy, and R0 resection was reached after operation. In the future, the exploration of the conversion therapy and the application of PET-CT in the evaluation of efficacy are another meaningful research direction.

Some case reports have indicated the successful use of targeted drugs (such as trastuzumab) in conversion therapy of patients with gastric cancer (Choda et al., 2014; Yamamoto et al., 2014). The efficacy and safety of apatinib in palliative treatment of advanced gastric cancer have been supported by previous studies (Li et al., 2013; Li et al., 2016). A small number of clinical and preclinical studies have shown that antivascular drug combined with chemotherapy can significantly reduce the stage of solid tumors (Li and Wang, 2017; Zheng et al., 2018; Zhao et al., 2020). There is not a lot of literature reference about apatinib combined with chemotherapy in the treatment of advanced gastric cancer. In the patients evaluated in this study, with the combination of targeted therapy and chemotherapy, the tumor was significantly controlled, the tumor stage was decreased, and the progression-free survival time was prolonged after conversion surgery, thus prolonging the average survival time of patients. However, this study still has some limitations. Firstly, the study only included three patients in a single center. In order to verify the effect of the conversion therapy, more rigorous clinical studies are needed to be carried out. Secondly, the apatinib is currently only used in the third-line treatment of gastric cancer. This article lacks research on the mechanism of apatinib combined with SOX as a conversion therapy. In the future, we will carry out more basic research to illustrate in most cases

This study provides a new idea for the treatment of advanced gastric cancer. The efficacy of targeted therapy combined with chemotherapy in patients with advanced gastric cancer still needs a large sample and multi-level randomized control study.

## Data Availability Statement

The original contributions presented in the study are included in the article/supplementary material; further inquiries can be directed to the corresponding authors.

## Ethics Statement

The patients/participants provided their written informed consent to participate in this study. The studies involving human participants were reviewed and approved by The Ethics Committee of The people’s Hospital of Zhejiang Province (Hangzhou, China). Written informed consent was obtained from the individual(s) for the publication of any potentially identifiable images or data included in this article.

## Author Contributions

DJ, ZC, and LY wrote the main manuscript. LY, ZC made contribution to conception and design. YX, JJ, YC, MW, and MY performed the data collection. All authors contributed to the article and approved the submitted version.

## Funding

This work was supported by the National Natural Science Foundation of China (No. 81772575, 81972455, 81802623).

## Conflict of Interest

The authors declare that the research was conducted in the absence of any commercial or financial relationships that could be construed as a potential conflict of interest.

## Abbreviations

PCR, polymerase chain reaction; PFS, progression-free survival; OS, overall survival; FIL, fondazione italiana linfomi; CR, complete remission; PR, partial remission; PET, positron-emission tomography; ESMO, European Society for Medical Oncology.
